# The Tick as a Cause of Cattle Fever

**Published:** 1892-03

**Authors:** 


### THE TICK AS A CAUSE OF CATTLE FEVER (?)

In the last issue of the *Texas Sanatarian* it is stated that at a recent meeting in Austin, of the Stock Raiser's Convention, "Mr. Rogers believed that Texas cattle fever is caused by ticks." A resolution was adopted, referring the subject to the directors of the Agricultural Experiment Station (with a specimen of tick [?] ), and with request to investigate and report.

The JOURNAL, through its own undergound telegraph system, is in receipt of an outline of the report the directors will make, far ahead of any other journal, and thus rejoices in what ye newspaper men call a "scoop." The subject being one full of interest as demonstrating the progress of sanitation in the interest of the *live stock* of Texas, for whose protection so much has already been done, we hasten to lay it before our readers:

YE REPORT.

[Advance sheets for DANIEL'S TEXAS MEDICAL JOURNAL by "our own correspondent."]

A. & M. COLLEGE OF TEXAS, EXPERIMENT STATION NO. 1,
OFFICE OF MEDICAL AND SANITARY DIRECTORS,
MARCH 10, 1892.

*To the Honorable Association of Cattlemen of Texas:*

GENTLEMEN:—In pursuance of request from your honorable body to investigate the relation as causative agent, of specimen submitted, and the disease called Texas cattle fever, we beg to report that a large number of experiments have been made at this station, both upon the specimen furnished us, and upon wild, wooly and untamed specimens, procured in the woods. We find that the "specimen" is a well fed, and well developed *ba[h]cillus*, and belongs to an unclassified group of the *genus ticque*; probably the *t. Texiensis robusta.* It is unicellular, binocular, gregarious, omniverous and persistent. It does not stain to any agent, but will *stay in* indefinitely when once burrowed into a "suitable nidus," e. g.—a cow's hide. Like the Klebs-Sœffler bacillus, it does not enter the system; never penetrates deeper than the *cutis-vera*, or true skin, but ejects a fluid of alkaline reaction and bitter taste, which fluid is taken up by the blood and lymph vessels, and carried into the general circulation. This fluid secretion, it is believed, is the poison which causes the fever, and there is reason to believe also, from experiments made, that it is the cause of those sudden impulses or freaks of cattle called "stampedes," which have never been accounted for. It is claimed by some, and the directors would say the claim is not without color (neither is the secretion); that the *ticque Texiensis robusta*, having been known to chew tobacco, as all other Texans do,—the secretion is most probably *tobăcco juice.* This, however, is, at present, *sub-judice.*

It would follow, therefore, as a rational deduction from the premises; that to prevent the fever, we must eliminate or destroy the cause, and the ba[h]cillus *t. Texiensis robusta* being assumed to be the cause we must prevent its attacks.

Experiments were made with a number of germicides and anti-sep-*ticks*, and, as was to be expected, the bi-chloride possessed the highest anti-septic(k) powers. Hot water (boiling point), ranked next; but this agent must be excluded from the fact that it cannot be applied *in looc*; its cosmetic effect is bad, as it destroys not only the bacillus, but the hair and hide also, thus rendering the cattle unsightly. The same objection applies to a very low temperature. To destroy the bacillus, freezing is necessary; and it was found that, although most Texas cattle are used to cold weather—from a life habit of sleeping out of doors, they do not bear freezing well in the live state. Before being refrigerated the animal should be killed.

As cattle are exposed to the ravages of the bacillus, both on the range and on the trail to northern markets or feeding grounds—the woods being full of them, the directors beg leave to recommend the following rules as affording the best and surest means of protection:

Each cattle should be thoroughly washed with soft water (the lime in some Texas waters having been found not to agree with well-bred and aristocratic cattle), and a good article of soap. (From numerous experiments the directors are convinced that Pears' soap is best, "on account of its peculiar and characteristic properties." See ad. in *Cosmopolitan*.) After being well dried by tissue paper napkins, rendered thoroughly antiseptic, a bichloride solution (1-500) should be applied to the entire external surface of each cattle, and as a further protection of those parts which come in contact with the grass and underbrush, the "habitat" of the bacillus, it is recommended to envelop the lower part of each leg, from the hoof to the knee, with antiseptic gauze roller bandage. If cosmetic effect be a desideratum, in case the cattle are to visit Chicago or St. Louis, and the owner would like them to appear well in society, these bandages may be selected with this view. Alternate stripes of red white and blue, not only produce a stunning effect when artistically wrapped around the legs of a young Texas cow, but it would be hailed as another evidence that Texas recognizes the fact that the war is over; taken as an overture to a restoration of brotherly love; a bridging of the bloody chasm, as it were. Similarly, such bandages if applied to the horns may be very effective, both in keeping off the dread invaders and in displaying the loyalty and patriotism of the Texas cattle kings.

Respectfully submitted,

SEPTUS C. MIER, M. D , Director.

